# Current Sensorless Based on PI MPPT Algorithms

**DOI:** 10.3390/s23104587

**Published:** 2023-05-09

**Authors:** Moacyr A. G. de Brito, Guilherme M. S. Martines, Anderson S. Volpato, Ruben B. Godoy, Edson A. Batista

**Affiliations:** Electrical Engineering Department, Faculty of Engineering, Architecture and Urbanism and Geography—FAENG, Federal University of Mato Grosso do Sul—UFMS, Costa e Silva Avenue, Campo Grande 79070-900, MS, Brazil; guilherme.martines@ufms.br (G.M.S.M.); anderson.volpato@ufms.br (A.S.V.); ruben.godoy@ufms.br (R.B.G.);

**Keywords:** controllers, MPPT, reduced cost, sensorless

## Abstract

This paper presents novel current sensorless maximum-power point-tracking (MPPT) algorithms based on compensators/controllers and a single-input voltage sensor. The proposed MPPTs eliminate the expensive and noisy current sensor, which can significantly reduce the system cost and retain the advantages of the widely used MPPT algorithms, such as Incremental Conductance (IC) and Perturb and Observe (P&O) algorithms. Additionally, it is verified that the proposed algorithms, especially the proposed Current Sensorless V based on PI, can reach outstanding tracking factors (TFs) such as the IC and P&O based on PI algorithms. In this sense, the insertion of controllers inside the MPPT gives them adaptive characteristics, and the experimental TFs are in the remarkable range of more than 99%, with an average yield of 99.51% and a peak of 99.80%.

## 1. Introduction

Sustainability is the core of electricity production, management, and consumption. Conventional primary energy sources such as coal and oil are capacity-limited. Additionally, they emit unsafe substances throughout the course of their usage, engendering hazardous impacts on both the environment and living beings [1]. The use of renewable energy sources becomes inspiring as long as they are inexhaustible on a human time scale and emit very low levels of pollutants; in fact, most have zero emissions during operation [2].

Among the vast number of renewable energy sources, solar photovoltaic (PV) technology has exhibited tremendous growth due to the enormous availability of energy from the sun in several countries, coupled with a reduction in the cost of installing and maintaining PV systems over time. Installed solar power capacity grew from 100 GW in 2012 to 1 TW in 2022; this represents a remarkable reduction in carbon dioxide emissions. In addition, the global capacity is predicted to reach 18 times its current level, exceeding 8 TW by 2050, representing more than 25% of the world’s energy matrix [3].

One issue with PV usage is the reduced quantity of energy converted, i.e., the tangible efficiency of the photovoltaic conversion. For example, some of the best commercial photovoltaic modules have an average yield of approximately 19–22%, which could hinder the dissemination of this technology [4]. In laboratory environments, in the case of multi-junction cells, it is already possible to observe energy yields of up to 47%. However, it is not easy for such modules to reach the market [5]. However, the amount of energy converted from the PV module can be augmented using a power electronics converter controlled via a maximum power point tracking algorithm (MPPT) [6,7,8]. These converters act dynamically by varying the load impedance “seen” by the module through voltage, current, and duty cycle variations to promote maximum power transfer [9].

In that sense, a great number of MPPT techniques have been introduced, discussed, and tested, such as perturb and observe (P&O) [10,11,12], resistance perturbation and observation (RP&O) [13], incremental conductance (IC) [14,15,16,17], the beta method [18], ripple correlation [19,20], systems oscillation [21,22], MPP locus characterization [23], reinforcement learning [24], sliding mode [25], and compensator-based methods [7,26]. Among these approaches, the P&O and IC based on the PI MPPT algorithms demand special attention because they offer outstanding performance and high speeds during initialization procedures [7,26].

Maximum tracking efficiency is commonly achieved using algorithms that use two sensors, one for voltage and another for current. The quality index that measures the energy harvest is referred to as the Tracking Factor (TF), which is above 98% for optimized MPPT systems that utilize both sensors. The TF measures the energy that the MPPT algorithm can convert [7]. It is essential to highlight that the scenarios used to test the MPPTs should have different irradiance and temperature variations to produce varying power profiles.

However, the use of current sensors leads to an increase in system costs and the need to improve the signal conditioning and control system immunity performance due to the noise inherent to the current waveform. The adoption of bandwidth reduction and filter insertions (analog and digital) may not always be straightforward, as they may disrupt the system’s MPP [27].

To reduce the costs of MPPT systems, it is desirable to avoid current sensors. Nevertheless, the exclusion of this sensor, and consequently, the current signal, leads to a reduction in the TF to a range of 90–92% for well-tuned algorithms [7].

Recently, many sensorless MPPT techniques have been proposed. According to which sensors are eliminated, the sensorless MPPT techniques can be classified into two distinct groups: the Current Sensorless (CS) group [28,29,30,31], which eliminates the current sensors, and the Voltage Sensorless group (VS) [32,33,34,35], which eliminates or reduces the number of voltage sensors.

Although the costs are reduced when using only a voltage sensor, one should consider that during irradiance changes, the current varies significantly more than the PV voltage at the MPP [7]; thus, CS methods are a more attractive option.

Considering the practical aspects of a CS method, a point that should be highlighted is that the current in a power electronics converter can be estimated according to its duty cycle, as well as the relationship between the input and output voltages or the input voltage and its static gain [9,36].

Based on these considerations, this paper proposes novel current sensorless MPPT algorithms that use only one single-input voltage sensor and incorporate compensators within the algorithm. This eliminates the need for expensive current sensors, which can significantly reduce the system costs while maintaining the advantages of the widely used MPPT algorithms such as IC and P&O. The absence of a current sensor is mitigated from employing a mathematical estimation of the PV current. Furthermore, the inclusion of a compensator allows the algorithms to work as a control system, presenting adaptive characteristics to reach outstanding TFs in the range of 99%, close to the performance of P&O and IC based on PI.

## 2. PV Modeling

**Figure 1:**
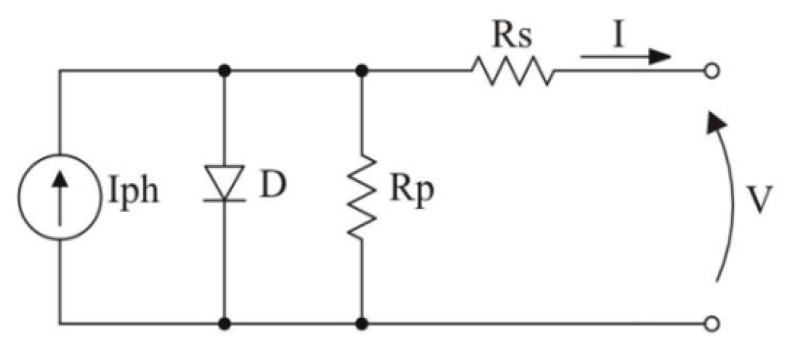
Fundamental PV cell model.

The equivalent circuitry of a fundamental PV cell is presented in Figure 1. As noted, this model includes a current source $I_{ph}$ that is anti-parallel with a diode. The non-idealities are characterized by the inclusion of the resistances $R_{s}$ (series resistance) and $R_{p}$ (parallel resistance). This model is simple and highly accurate [37].

The derived PV simulation model is based on the output current of one PV fundamental cell (I), and its mathematical modeling is presented in Equation (1). Then, as the cells are series-connected in a PV module, this current is the real output current, and the voltage is the summing of the individual voltages, which is expressed as follows:

(1) $$I=I_{ph}-I_{r}\left[e^{\frac{q\left(V+IR_{s}\right)}{\eta kT}}-1\right]-\frac{V+IR_{s}}{R_{p}},$$

where V represents the output PV voltage of one PV cell, $I_{ph}$ is the photocurrent, $I_{r}$ is the saturation current, q is the electrical charge (1.6 × 10^−19^ C), η is the p-n junction quality factor, k is the Boltzmann constant (1.38 × 10^−23^ J/K), and T is the temperature (K).

The non-linearity and current dependency can be observed in Equation (1). So, Equation (1) can be adjusted to present a null root when current I approaches the real PV current. Therefore, Equation (1) became Equation (2) in the function of the own PV current since

(2) $$f\left(I\right)=I_{ph}-I-I_{r}\left[e^{\frac{q\left(V+IR_{s}\right)}{\eta kT}}-1\right]-\frac{V+IR_{s}}{R_{p}}.$$

Then, the current I, with a null initial value, is used in an iterative process that approximates Equation (2) of its root, obtained via any numerical method. Here, the Newton–Raphson method was adopted according to Equation (3), which seeks zero for the differentiable function as follows:

(3) $$x_{n+1}=x_{n}-\frac{f\left(x_{n}\right)}{f^{\prime }\left(x_{n}\right)}.$$

Thus, the derivative of Equation (2) is presented in Equation (4) as follows:

(4) $$f\left(I\right)=-I-I_{r}\left[e^{\frac{q\left(V+IR_{s}\right)}{\eta kT}}\right]\frac{qR_{s}}{\eta kT}-\frac{R_{s}}{R_{p}}.$$

With Equations (1)–(4), an embedded function was created to simulate the PV module in the MATLAB/Simulink^®^ environment. The model was built as a voltage-dependent current source to feed a decoupling capacitance that stores the injected current from the PV. Any power electronics converter could be attached to this capacitance to extract power. The ones that have an input inductance are preferable to minimize the input ripples. The electrical parameters of the PV are presented in Table 1.

**Figure 2:**
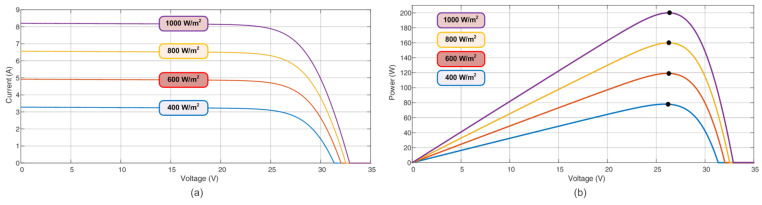
PV power characteristics for different irradiance levels at 25 °C (298 K): (**a**) Current; (**b**) Power Profile—PxV. The black dots represent the real MPP.

**Figure 3:**
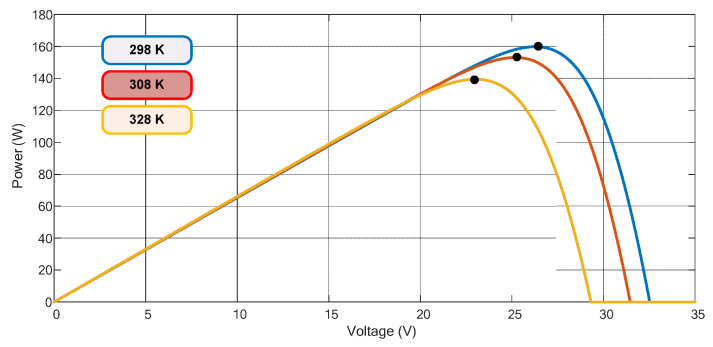
PV power characteristics for different temperature levels. Irradiance is considered 800 W/m^2^. The black dots represent the real MPP.

Figure 2 and Figure 3 depict the power characteristics of the analyzed PV module, considering solar irradiation (W/m^2^) and temperature changes (K). These curves show the PV’s non-linear characteristics and how they are intensely influenced by climate variations.

## 3. Proposed MPPT Mathematical Modeling

In this section, the mathematical modeling of the novel MPPT algorithms is derived. Considering a boost DC–DC converter as the interface converter for the PV module, according to Equations (5)–(7), one can obtain the static gain (G) as a function of the PV voltage ($V_{PV}$) and the DC Bus voltage ($V_{BUS}$) [9] as follows:

(5) $$G=\frac{V_{BUS}}{V_{PV}}=\frac{1}{1-D},$$

(6) $$G=\frac{I_{PV}}{I_{BUS}}=\frac{1}{1-D},$$

(7) $$I_{PV}=\left(\frac{1}{1-D}\right)I_{BUS}.$$

By using Equation (7) and considering that the averaged consumed power is drained via a load resistance ($R_{L}$), one can find Equation (8):

(8) $$I_{PV}=\left(\frac{1}{1-D}\right)\frac{V_{BUS}}{R_{L}}.$$

Using Equation (5), it is possible to rewrite Equation (8) as follows:

(9) $$I_{PV}=\frac{V_{PV}}{R_{L}}{\left(\frac{1}{1-D}\right)}^{2}.$$

The power extracted from the PV module is calculated from its input as follows:

(10) $$P=V_{PV}I_{PV}.$$

Substituting Equation (10) into Equation (9) yields the following:

(11) $$P=\frac{V_{PV}{}^{2}}{R_{L}}{\left(\frac{1}{1-D}\right)}^{2}.$$

In the MPP, the derivative of power versus voltage $\left(\frac{dP}{dV}\right)$ is zero; thus,

(12) $$\frac{dP}{dV}=\left[\frac{V_{PV}{}^{2}}{R_{L}}{\left(\frac{1}{1-D}\right)}^{2}\right]/dV_{PV},$$

(13) $$\frac{dP}{dV}=\frac{2V_{PV}}{R_{L}}{\left(\frac{1}{1-D}\right)}^{2}+\frac{V_{PV}{}^{2}}{R_{L}}\frac{d{\left(\frac{1}{1-D}\right)}^{2}}{dV_{PV}},$$

(14) $$\frac{dP}{dV}=\frac{V_{PV}}{R_{L}}{\left(\frac{1}{1-D}\right)}^{2}\left[2+V_{PV}{\left(1-D\right)}^{2}\frac{d{\left(\frac{1}{1-D}\right)}^{2}}{dV_{PV}}\right],$$

In digital applications, one can approximate the derivative by its variation over time (∆). Thus, it is possible to obtain the constraint to reach the MPP, denoted as $MPPT_{cons}$, as Equation (15):

(15) $$2+V_{PV}{\left(1-D\right)}^{2}\frac{∆{\left(\frac{1}{1-D}\right)}^{2}}{∆V_{PV}}=MPPT_{cons}=0.$$

During the algorithm exploitation in the searching space for the MPP finding, according to the typical PV curves (P × V and I × V), one may verify the constraints.

(16) $$\left\{\begin{array}{c} MPPT_{cons}>0\to Decrease\;D\;or\;increase\;V_{FV}\\ MPPT_{cons}<0\to Increase\;D\;or\;decrease\;V_{FV} \end{array}\right..$$

The novel MPPT algorithms use the condition of Equation (15), which embeds the constraints of Equation (16), to reach the MPP and directly varies the duty cycle or the PV input voltage. Condition (15) is achieved through a compensator or a controller, which can be any type of compensator. In this study, we have adopted integral and proportional plus integral controllers (PI controllers). Therefore, the proposed algorithms are named Sensorless D or Sensorless V based on PI, which varies D or $V_{PV}$, respectively.

**Figure 4:**
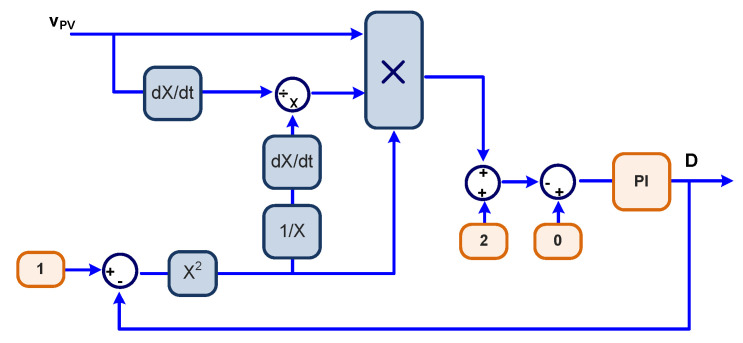
MATLAB/Simulink^®^ Sensorless D MPPT based on PI model.

**Figure 5:**
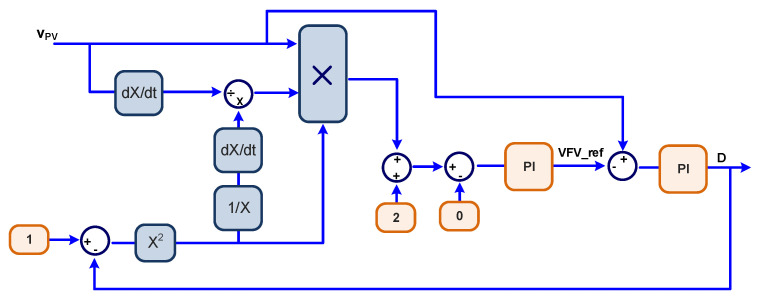
MATLAB/Simulink^®^ Sensorless V MPPT based on PI model.

The MATLAB/Simulink^®^ block diagram to implement the Sensorless D is depicted in Figure 4, whereas the Sensorless V MPPT is presented in Figure 5.

## 4. Controlled MPPT Transfer Functions 

Considering the PV module (or PV module association) as a current source ($I_{pv}$) and that the power control loop regulates the $C_{\mathrm{BUS}}$ voltage towards an average value ($V_{BUS}$), it is possible to obtain the input to control transfer function for the DC–DC MPPT control.

Observe that this algorithm can control the MPP in a Boost converter feeding a stand-alone load or feeding the DC Bus of a Voltage Source Inverter once, as mentioned, the inverter regulates the DC Bus voltage through the power control loop in an almost constant value [38].

If only the Boost converter is assumed, the designer may consider a resistive load ($R_{L}$) to produce the desired output voltage ($V_{BUS})\;$ at the MPP. In such a sense and in both cases, the input decoupling capacitance ($C_{in}$) and the input inductance ($L_{in}$) are extremely important for the MPP harvesting, i.e., they guide the MPPT dynamics.

**Figure 6:**
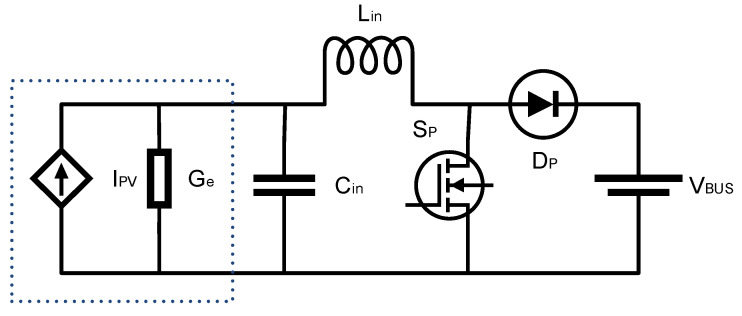
PV source plus DC–DC converter for MPPT modeling.

The operation at the maximum power point is represented via the PV equivalent conductance ($G_{e}$) insertion, as one can verify in Figure 6. 

So, Equations (17) and (18) are found considering the average state equations as follows:

(17) $$I_{pv}-i_{Lin}-i_{Ge}=C_{in}\frac{dV_{Cin}}{dt},$$

(18) $$v_{Cin}-\left(1-d\right)V_{BUS}=L\frac{di_{Lin}}{dt},$$

where the capital letters represent the constant values, and the small letters represent the variables.

After applying small-signal analysis, Laplace transforms, and some mathematical manipulations, one can derive Equation (19). Equation (19) characterizes how the input voltage $v_{cin}=v_{pv}$ varies when the Boost duty cycle is varied. Thus, by controlling the duty cycle of the power electronics converter, the PV voltage can be controlled to reach the MPP. The inverse association between the input voltage and the converter duty cycle is verified in Equation (19) as follows:

(19) $$G_{vCin_{d}}\left(s\right)=\frac{v_{Cin}\left(s\right)}{d\left(s\right)}=\frac{-V_{BUS}}{\left(1+sL_{in}G_{e}+s^{2}L_{in}C_{in}\right)}.$$

The negative sign in Equation (19) is incorporated in the models shown in Figure 4 and Figure 5. The dynamics of the PV itself can be neglected once the PV reproduces almost instantaneously the power variations when submitted to irradiation and temperature changes. Therefore, based on Equation (10), one can derive the power to the voltage transfer function.

(20) $$G_{PV}=\frac{dP}{dV_{PV}}=I_{PV}.$$

Using the aforementioned Sensorless D MPPT algorithm, it is necessary to adapt Equations (19) and (20) for use in a single control loop. Thus, the input power P is changed by varying the duty cycle D; this is accomplished via Equation (21) to tune the controller as follows:

(21) $$G_{Pd}\left(s\right)=G_{vCin_{d}}\left(s\right).G_{PV}=\frac{-V_{BUS}.I_{PV}}{\left(1+sL_{in}G_{e}+s^{2}L_{in}C_{in}\right)}.$$

For the Sensorless V MPPT, it is necessary to use both Equations (20) and (19) independently. Equation (20) is used to derive the controller which will generate the reference for the PV voltage (${\mathrm{VFV}}_{\mathrm{REF}}$ in Figure 5), while Equation (19) is used to derive the controller to produce the duty cycle D. This results in a two-loop control MPPT process.

**Figure 7:**
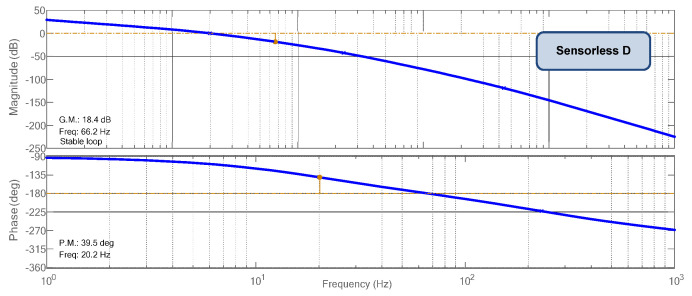
Compensated Bode diagrams for the Sensorless D with $C_{Pd}$ *(s)*.

**Figure 8:**
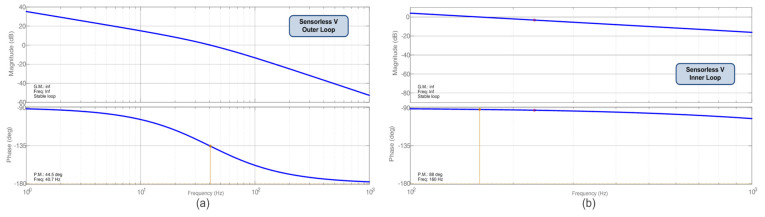
Compensated Bode diagrams for the Sensorless V (**a**) with $C_{PV}$ *(s)* and (**b**) with $C_{Vd}$ *(s)*.

One can verify in Figure 7 and Figure 8 the Bode diagrams of module and phase to accomplish the input capacitor’s voltage regulation (MPP finding). In Figure 7, one can verify the Bode plots for the Sensorless D MPPT obtained using Equation (21); in Figure 8a,b, the Bode plots for the Sensorless V MPPT, through Equations (19) and (20). Table 2 summarizes the parameters of Boost + PV for the obtainment of the MPPT controllers. The voltage sensor gain is assumed to be unitary.

An integral plus filter compensator was chosen to perform the task for the Sensorless D MPPT. The acquisition frequency is 1 kHz, and the cut-off frequency of the filter is 20 Hz, the same as the desired MPPT velocity, i.e., the final Bode crossover frequency is expected to be in the range of 20 Hz.

It is possible to verify the crossover frequency and stability margins in Figure 7, at 20.2 Hz and 40 degrees, respectively. Equation (22) presents the proposed controller.

(22) $$C_{Pd}\left(s\right)=\frac{0.25}{s}\frac{2\pi 20}{s+2\pi 20}.$$

An integral plus filter compensator and a PI controller were selected as controllers for the Sensorless V MPPT. The acquisition frequency is also 1 kHz, and the filter cut-off frequency is 40 Hz, the same as the desired MPPT velocity for the power loop. As this approach is even more stable, it is possible to increase the outer loop velocity. The voltage loop, which represents the inner loop, is expected to work in the range of 100–200 Hz, faster than the outer loop.

It is possible to verify the crossover frequency and stability margins in Figure 8, as 40.7 Hz and 40 degrees, and 160 Hz and 88 degrees, for the outer and inner loops, respectively. Equation (23) presents the proposed controller for the power loop (outer loop), and Equation (24) presents the proposed controller for the voltage loop (inner loop) as follows:

(23) $$C_{PV}\left(s\right)=\frac{50}{s}\frac{2\pi 40}{s+2\pi 40},$$

(24) $$C_{Vd}\left(s\right)=\frac{0.006\left(s+1450\right)}{s}.\;$$

## 5. Results and Discussion

Among the performance measures, the transmitted energy is essential for the usage of the PV module as an energy source. This important measure is the TF, tracking factor, as previously mentioned, which is the percentage of the available energy that was converted [7]. The ripple voltage in a steady state has vital importance as there is a limit of ripple for the PV system to remain effective at the MPP. For the algorithm to reach 98% of the power extracted, the ripple voltage at the MPP should not exceed 8.5% in a steady state [39].

**Figure 9:**
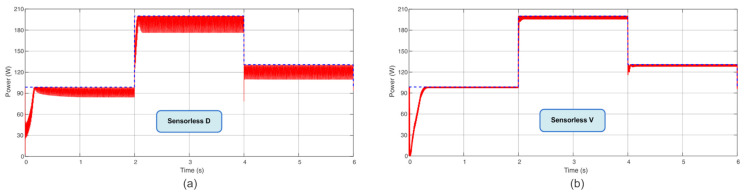
Power extracted (**a**) using Sensorless D algorithm and (**b**) using Sensorless V algorithm.

**Figure 10:**
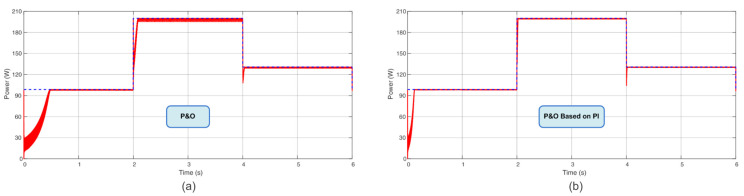
Power extracted (**a**) using the P&O algorithm and (**b**) using the P&O based on PI algorithm.

**Figure 11:**
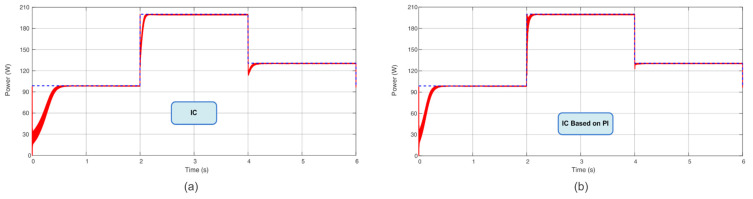
Power extracted (**a**) using the IC algorithm and (**b**) using the IC based on PI algorithm.

Following the power profile used in [7], with steps of irradiance and temperature every 2 s, in a total time of 6 s, this present work compared the TF of the proposed Sensorless D and Sensorless V with the P&O, P&O based on PI, IC, and IC based on PI. According to these results, the novel proposed MPPT Sensorless V reached almost 98% of TF in these conditions. The computed TF considers the initialization procedure (initiation from almost non-power). This is a remarkable finding since it is almost close to the TF of the IC and P&O based on PI methods but without the usage of the current sensor. Table 3 summarizes the obtained TFs, and Figure 9, Figure 10 and Figure 11 show the extracted power using the aforementioned algorithms.

The algorithms are tuned similarly to a control system with controllers being integrated into the system to allow the MPPT to have the desired crossover frequency and phase margin. In this way, as the response of the MPPT is related to the input error and its tuning, the algorithm exhibits a fast response during power variations (due to environmental conditions) and in a steady state, the algorithm exhibits reduced fluctuation.

**Figure 12:**
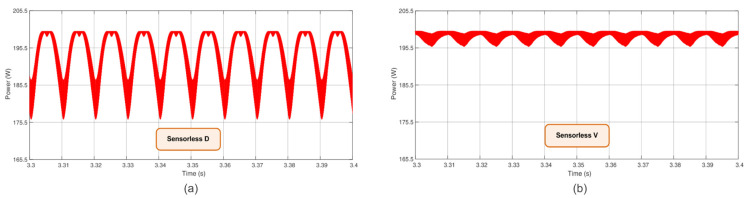
Power ripple (**a**) for the Sensorless D at the MPP and (**b**) for the Sensorless V at the MPP.

Considering the need to evaluate the power ripple in a steady state, Figure 12a,b presents the power ripple for the proposed Sensorless D and Sensorless V based on PI algorithms, respectively. One can verify that the Sensorless V presents much less power fluctuation than the Sensorless D. The power ripple for the Sensorless D is in the range of 12.25%, while the Sensorless V presents only 2.25%, i.e., it is in the true MPP. 

**Figure 13:**
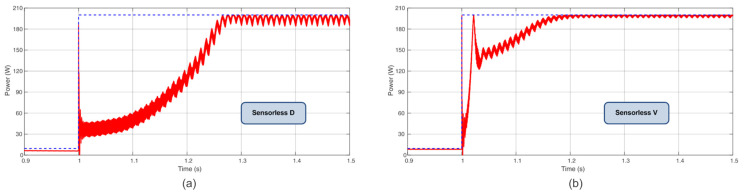
Initialization (**a**) for the Sensorless D and (**b**) for the Sensorless V.

The initialization performance was tested considering a sudden power change from 10 W to 200 W. The Sensorless D achieved a steady state at 280 ms, while the Sensorless V achieved the same condition at 180 ms. Thus, the proposed algorithms show a fast dynamic response that can be seen in Figure 13a,b.

**Figure 14:**
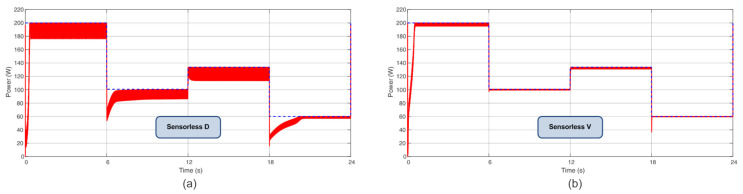
Power extracted considering Profile I (**a**) for the Sensorless D and (**b**) for the Sensorless V.

**Figure 15:**
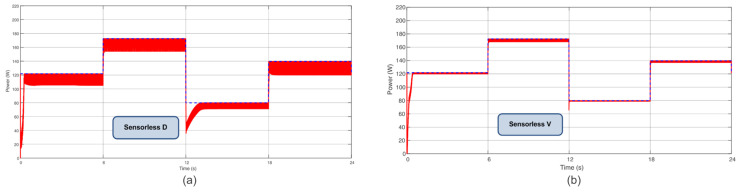
Power extracted considering Profile II (**a**) for the Sensorless D and (**b**) for the Sensorless V.

Other power profiles were applied to test the effectiveness of the proposed algorithms in different scenarios. The first and second profiles consider four steps of power with varying irradiance and temperature, as shown in Table 4. These profiles are similar to [26]. For Profile I, the Sensorless D reached 94.10%, and Sensorless V reached 98.85%; when considering Profile II, the Sensorless D reached 95.28%, and Sensorless V reached 99.05%. Figure 14 and Figure 15 show power extraction. Compared with [26], the proposed Sensorless V based on PI achieved analogous performance to the P&O based on PI, which achieved 99.17% and 99.32% for profiles similar to I and II, respectively.

**Figure 16:**
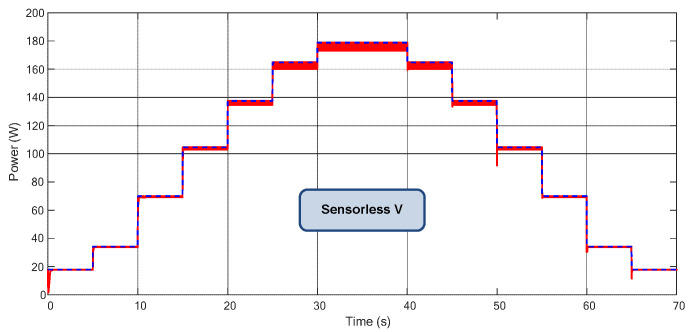
Power extraction for the Sensorless V considering a typical daily power profile.

Finally, in Figure 16, a daily power profile emulating the temperature and irradiance changes during a typical PV day from 6 AM to 6 PM is shown for the Sensorless V based on PI MPPT algorithm. In such conditions, the proposed MPPT reached 99.25% of all available power which is a remarkable TF for an MPPT with only one voltage sensor.

Observing the MPPT Sensorless V TF quality, a power electronics converter was built and attached to a solar array emulator (E4350B—from Agilent) to experimentally verify the aforesaid findings. An acquisition management system was used in the experiments to acquire the power waveforms and compute dynamically the TF. This management system is a PC user-friendly graphical interface that was implemented by the corresponding author and can be verified in [7].

**Figure 17:**
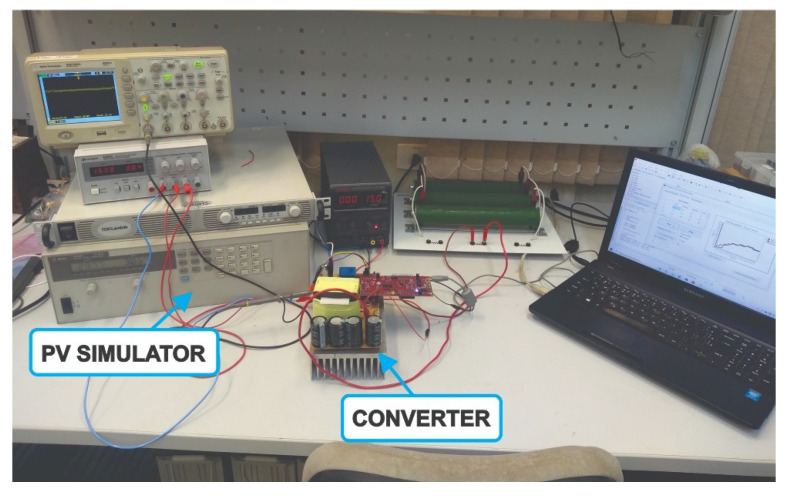
Experimental setup to evaluate the Current Sensorless V based on PI MPPT.

The converter specifications follow Table 2 parameters, and the Current Sensorless V based on the PI MPPT algorithm was digitally implemented in the Launchpad F28379D, from Texas Instruments. In Figure 17, the experimental setup can be seen.

**Figure 18:**
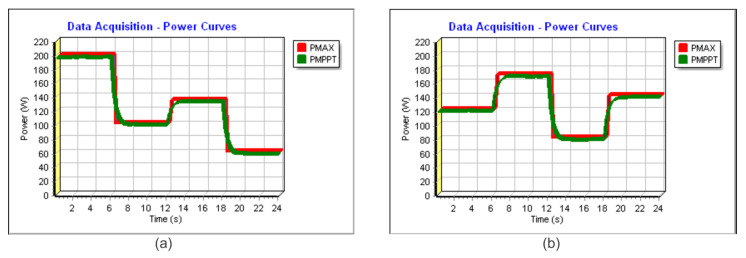
Experimental power extracted for the Sensorless V—total time 24 s (**a**) considering Profile I and (**b**) considering Profile II.

**Figure 19:**
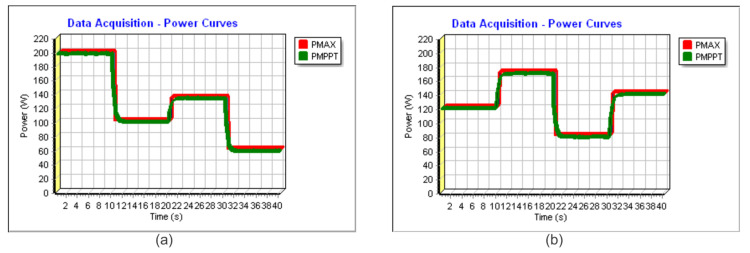
Experimental power extracted for the Sensorless V—total time 40 s (**a**) considering Profile I and (**b**) considering Profile II.

In Figure 18a and Figure 19a, the power extracted can be found in similar conditions to Profile I. In Figure 18a, the time step is 6 s, and, in Figure 19a, the time step is 10 s. In red, it can be verified the theoretical maximum available power (PMAX) and, in green, the extracted power (PMPPT). In such conditions, for Figure 18a and Figure 19a, the algorithm achieved a TF of 99.32% and 99.48%, respectively.

Following Profile II, the energy harvesting can be verified using Figure 18b and Figure 19b. The achieved TFs were 99.43% and 99.58%, respectively.

**Figure 20:**
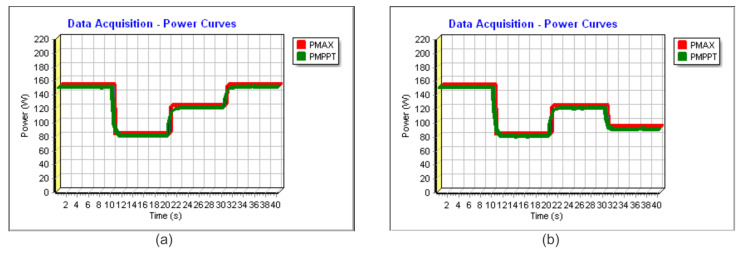
(**a**) Experimental power extracted for the Sensorless V—total time 40 s. (**b**) Experimental power extracted for the Sensorless V—total time 40 s.

**Figure 21:**
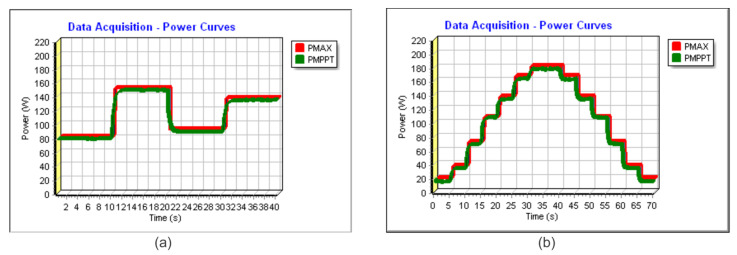
(**a**) Experimental power extracted for the Sensorless V—total time 40 s. (**b**) Power Extraction for the Sensorless V considering a typical daily profile, similar to profile of Figure 16—total time 70 s.

Different tests were conducted to continuously verify the TF of the proposed Current Sensorless V based on PI MPPT. The results can be verified in Figure 20a,b and Figure 21a. The obtained TFs were 99.80%, 99.68%, and 99.61%, respectively.

A daily power profile similar to Figure 16 was verified experimentally and depicted in Figure 21b. The calculated TF was 99.18%.

It is noteworthy that the reduced power fluctuation in the energy extraction using the proposed approach greatly increases energy extraction. The obtained TFs were in the range of 99%. Among the tests, the average TF was 99.51%.

According to the tests, the proposed Sensorless V MPPT based on PI is an interesting alternative to the existing MPPT algorithms.

A comparison with some existing sensorless MPPT algorithms regarding the tracking factor under experimental conditions can be observed in Table 5. It can be noticed the high quality of the proposed Sensorless V based on the PI MPPT algorithm. For comparison purposes, the average TF was inserted; however, one can also consider that a peak TF of 99.8% was achieved, higher than all experimentally validated MPPTs of Table 5.

In addition, the algorithm was evaluated regarding the time required to complete its task. The time is about 580 ns. As the acquisition frequency is 1 kHz, the developer has almost the entire cycle to implement the other converter functionalities, and, thus, it can be seen how minimal the MPPT burden is for the microcontroller concerning the total available time. 

## 6. Perovskite Solar Cells

Additionally, as the proposed MPPT produces less power fluctuation in a steady state, it can be used to help extract power from Perovskite Solar Cells (PSCs), as conventional MPPTs do not function efficiently in such cells. The PSCs naturally exhibit a slow response of current in the face of varying voltage resulting in large power oscillations. In this circumstance, inserting a control system inside the MPPT for tuning its velocity could help improve the extraction of power in PSCs. The analysis of MPPTs for PSCs is not in the scope of this manuscript because PSCs are not yet a commercially viable alternative. However, additional information regarding MPPT and PSCs can be found in [44], and hybrid PSCs are currently evolving to help increase their average life and performance.

## 7. Conclusions

Considering the need for reducing costs while maintaining the quality of power extraction, it is possible to predict the PV current using the fundamental equations of the power electronics converters. Additionally, it is also interesting to transform the MPPT algorithm into a control loop via the insertion of controllers and functions that will be minimized. In this context, this article has presented the novel MPPT algorithms—Current Sensorless D and Current Sensorless V based on PI—which use only an input voltage sensor and are optimized via employing compensators.

The Sensorless V algorithm presented exceptional performance and outstanding results in terms of initialization and power ripple in steady-state for all tested power profiles. It outperformed the well-established P&O and IC methods and achieved comparable results to the P&O and IC based on PI MPPTs. It is noteworthy that the proposed algorithms achieved a high tracking factor with fewer sensors, resulting in reduced costs.

## Figures and Tables

**Table 1:** PV Electrical Parameters.

Electrical Parameters	Values
Maximum Power	P_max_ = 200 Wp
Voltage at MPP	V_MPP_ = 26.3 V
Current at MPP	I_MPP_ = 7.61 A
Open Circuit Voltage	V_oc_ = 32.9 V
Short Circuit Current	I_sc_ = 8.21 A
Temperature Coefficient of I_sc_	α = 3.18 × 10^−3^ A/°C

**Table 2:** Parameters of the Boost Converter + PV.

Electrical Parameters	Values
Maximum Power	P_max_ = 200 Wp
Voltage at MPP	V_MPP_ = 26.3 V
Current at MPP	I_MPP_ = 7.61 A
Decoupling capacitance	C_in_ = 10 μF
Boost inductance	L_in_ = 2.5 mH
Boost load	R_L_ = 50 Ω
Conductance	G_e_ = 0.2894 s

**Table 3:** Tracking Factors.

Methods	Values
Sensorless D	94.65%
Sensorless V	97.61%
P&O	95.75%
P&O based on PI	98.75%
IC	95.85%
IC based on PI	98.68%

**Table 4:** Varying Power Profiles.

	**Profile I**	
**Irradiance**	**Temperature**	**Theoretical Power**
1000 W/m^2^	25 °C	200.01 W
500 W/m^2^	20 °C	100.79 W
700 W/m^2^	35 °C	133.68 W
300 W/m^2^	15 °C	60.08 W
	**Profile II**	
**Irradiance**	**Temperature**	**Theoretical Power**
600 W/m^2^	20 °C	121.74 W
900 W/m^2^	35 °C	172.47 W
400 W/m^2^	20 °C	79.80 W
700 W/m^2^	25 °C	139.62 W

**Table 5:** Experimentally validated Sensorless MPPT Tracking Factors.

Methods	Values
Constant Voltage [40]	79.50%
Short Circuit Pulse [40]	89.70%
Open Circuit Voltage [40]	93.70%
LCASF [32]	95.00%
LCA [32]	96.00%
Proposed Current Sensorless V	99.51%
FSCC [33]	97.50%
Current Sensorless [41]	92.10%
ASC-MPPT [42]	96.20%
Hybrid FOCV-SCAM [29]	99.70%
SC-MPC-MPPT [43]	99.40%

## Data Availability

Not applicable.
